# Balancing Surgical Innovation with Indications: A Multicenter Retrospective Comparison of Reduced-Port Distal Gastrectomy Using da Vinci SP Versus Multi-Port Robotic Platforms from the KLASS-13 Cohort

**DOI:** 10.3390/cancers18050823

**Published:** 2026-03-04

**Authors:** Jae Hun Chung, Hyoung-Il Kim, Sang-Hoon Ahn, Han Hong Lee, Yun-Suhk Suh, Yoo Min Kim, Young Suk Park, Sung Hyun Park, Chang Min Lee

**Affiliations:** 1 Department of Surgery, Pusan National University Yangsan Hospital, School of Medicine, Pusan National University, Yangsan 50612, Republic of Korea; 2 Department of Surgery, Gastric Cancer Center, Yonsei Cancer Center, Yonsei University College of Medicine, Yonsei University Health System, Seoul 03722, Republic of Korea; 3 Department of Surgery, Samsung Medical Center, Sungkyunkwan University School of Medicine, Seoul 06351, Republic of Korea; 4 Department of Surgery, Seoul St. Mary’s Hospital, College of Medicine, The Catholic University of Korea, Seoul 06591, Republic of Korea; 5 Department of Surgery, Seoul National University Bundang Hospital, Seoul National University College of Medicine, Seongnam 13620, Republic of Korea; 6 Department of Surgery, Korea University Ansan Hospital, Korea University College of Medicine, Ansan 15355, Republic of Korea

**Keywords:** conventional reduced-port robotic distal gastrectomy, gastric cancer, KLASS-13, robotic gastrectomy, reduced-port robotic gastrectomy, single-port robotic gastrectomy

## Abstract

Background: The da Vinci single-port reduced-port robotic distal gastrectomy (spRRDG) approach shows promise in enhancing surgical recovery while maintaining oncologic safety, but robust multicenter comparative data across diverse robotic platforms are lacking. We aimed to compare clinical outcomes between spRRDG and conventional RRDG (cRRDG) using Korean Laparoendoscopic Gastrointestinal Surgery Study-13 data. Methods: Clinicopathologic variables and perioperative outcomes concerning 820 patients who underwent curative RRDG with D1+ or D2 lymph node dissection (LND) (da Vinci spRRDG, *n* = 86; cRRDG, *n* = 734) were analyzed. We compared continuous variables using Student’s t- or Wilcoxon rank-sum tests, as appropriate, and categorical variables using χ^2^ or Fisher’s exact tests. Subgroup analyses were performed according to the extent of LND (D1+ vs. D2). Statistical significance was defined as *p* < 0.05. Results: spRRDG involved a longer operative time than cRRDG (227.06 ± 6.19 vs. 183.58 ± 2.18 min, *p* < 0.0001) and fewer retrieved LNs (rLNs) (36.38 ± 1.53 vs. 46.52 ± 0.66, *p* < 0.0001), but showed superior enhanced recovery after surgery (ERAS)-related outcomes, including shorter hospital stay (4.06 ± 0.23 vs. 5.95 ± 0.13 days), and earlier gas passage (postoperative day [POD] 2.24 ± 0.10 vs. 3.08 ± 0.04) and soft diet initiation (POD 1.59 ± 0.14 vs. 2.89 ± 0.07; all *p* < 0.0001). In subgroup analyses, the number of rLNs was lower in D1+ spRRDG (34.09 ± 1.58 vs. 44.36 ± 0.72, *p* < 0.0001), but remained above the oncologic threshold (≥16 LNs). In D2 dissections, no significant difference was observed (45.71 ± 3.69 vs. 53.30 ± 1.39, *p* = 0.1030). Faster postoperative recovery in spRRDG persisted after adjustment. Conclusion: spRRDG exhibited lower rLNs than cRRDG but remained within an oncologically acceptable range. Comparable complication rates and significantly improved ERAS outcomes suggest spRRDG is safe and feasible; however, its clinical application should remain limited to early gastric cancer until robust evidence from prospective studies emerges.

## Simple Summary

Robotic gastrectomy is increasingly used for gastric cancer because it provides enhanced visualization and precise instrument control. As robotic systems have evolved, surgeons have developed reduced-port techniques to further minimize surgical trauma. More recently, a single-port robotic platform has enabled most surgical steps to be performed through a single main incision, with the goal of improving postoperative recovery. However, it is important to ensure that reducing the number of ports does not compromise cancer-related safety. We analyzed data from a multicenter registry of 820 patients who underwent reduced-port robotic distal gastrectomy, comparing outcomes between the single-port robotic system and the conventional multi-port platform. Although the single-port approach was associated with longer operative time and fewer retrieved lymph nodes, lymph node counts remained above internationally accepted standards for accurate cancer staging. Patients who underwent a single-port approach experienced faster postoperative recovery without an increase in short-term complications. These findings suggest that single-port robotic distal gastrectomy is safe and feasible in carefully selected patients, and its use should currently be limited to early-stage gastric cancer pending further long-term evidence.

## 1. Introduction

Continuous progress in surgical expertise and the development of advanced laparoscopic instruments have facilitated the evolution of minimally invasive surgery (MIS) in gastric cancer surgery, progressing from the initial form of laparoscopic gastrectomy (LG) to totally laparoscopic procedures [1,2,3,4,5]. These advances have further enabled the reduction in the number of abdominal ports, prompting the adoption of reduced-port and, ultimately, single-port LG. Each successive step lowers abdominal wall trauma and contributes to reduced postoperative pain and quick patient recovery, expanding the possibilities and benefits of MIS. These approaches, supported by previous studies, have shown safety and feasibility comparable to those of conventional multi-port LG [6,7].

However, reduced-port laparoscopic gastrectomy raises technical concerns, particularly regarding the adequacy of lymphadenectomy. The possibility of an inappropriate lymphadenectomy is a primary challenge regarding reduced-port LG, as lymph node dissection (LND) represents a crucial procedure in the surgical treatment of gastric cancer [8]. Moreover, the lack of surgical assistance in reduced-port LG poses a significant challenge for surgeons. In particular, the introduction of the multi-channel trocar to reduce the number of port wounds has led to poor ergonomics with conventional laparoscopic instruments in the narrow rim of the umbilical port [9].

To address these limitations, robotic gastrectomy (RG) has emerged as a promising alternative as it offers a stable operative field, enhanced dexterity, and precise tissue handling [10]. Recent comparative studies suggest that robotic gastrectomy offers advantages in postoperative recovery and complications compared with conventional laparoscopic approaches, with some reports also indicating improved oncologic outcomes [11,12,13,14]. Moreover, with the advent and widespread adoption of robot-surgery systems, reduced-port robotic gastrectomy (REPROG) has been developed through diverse methods and platforms [15,16,17,18,19,20,21,22]. In most procedures, surgeons attempt to reduce the number of ports by docking two or more arms to a multi-channel trocar. However, the current application of REPROG does not involve inserting multiple cannulas into a single umbilical port, as the approach has not yet been widely adopted. Therefore, applying REPROG is limited by the challenges of incomplete counter-traction and assistance.

Recent technological innovations in robot-surgery platforms have further advanced the MIS scope of REPROG. The da Vinci single-port (SP; Intuitive Surgical Inc., Sunnyvale, CA, USA) system integrates a multi-jointed endoscope and three articulating instruments into a single cannula. Despite the theoretical and practical appeal of single-port RG, extant evidence has predominantly been confined to retrospective analyses from single institutions [23,24,25]. Consequently, robust multicenter comparative data across diverse robotic platforms remain lacking. To bridge this knowledge gap, we aimed to compare the clinical outcomes between REPROG using the da Vinci SP system and conventional multi-port robotic platforms, leveraging the comprehensive Korean Laparoendoscopic Gastrointestinal Surgery Study (KLASS)-13 database to conduct the most extensive multicenter retrospective cohort for REPROG. This study is reported in line with STROCSS guidelines [26].

## 2. Materials and Methods

### 2.1. Data Collection

Under the auspices of the Korean Gastric Cancer Association (KGCA), the KLASS initiated the REPROG registry in May 2023 as its 13th research project [27]. Surgeons who had performed at least one REPROG case were eligible for inclusion. REPROG was defined as an RG performed using ≤3 trocars. Data were retrospectively accrued starting from the initial REPROG case of each participating surgeon through December 2023. Nine surgeons from four institutions contributed data to the KLASS-13 registry. All participating surgeons were board-certified gastrointestinal surgeons with prior experience in robotic gastrectomy. Each surgeon had performed more than 100 robotic gastrectomy procedures before contributing to the registry. However, the adoption of the da Vinci SP platform for distal gastrectomy began relatively recently (2022–2023), and therefore the SP experience represented an early adoption phase for most centers.

Ethical approval for human research was granted by the Institutional Review Board of Korea University Medical Center Ansan Hospital (registration number: 2025AS0022), and the requirement for informed consent was waived owing to the study’s retrospective design.

### 2.2. Patient Selection

**Figure 1:**
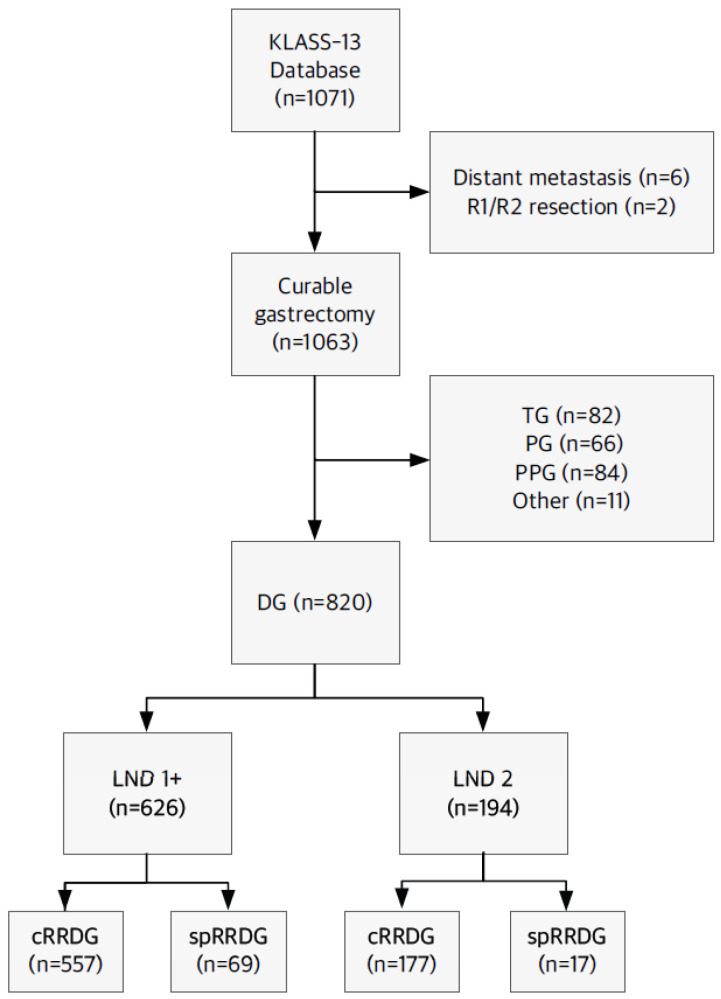
Study flow diagram of patient selection from the KLASS-13 registry.

In total, 1071 patients were initially identified from the KLASS-13 database. After excluding patients with distant metastasis (*n* = 6) and those who underwent R1 or R2 resection (*n* = 2), 1063 patients who underwent curative gastrectomy were screened. Patients who underwent total gastrectomy (TG, *n* = 82), proximal gastrectomy (PG, *n* = 66), pylorus-preserving gastrectomy (PPG, *n* = 84), or other procedures (*n* = 11) were excluded, yielding 820 patients who received distal gastrectomy (DG) (Figure 1).

Patients were categorized according to the extent of lymph node dissection (LND 1+ or LND 2), followed by comparison between the cRRDG and the spRRDG. Abbreviations: cRRDG, conventional reduced-port robotic distal gastrectomy; DG, distal gastrectomy; LND, lymph node dissection; PG, proximal gastrectomy; PPG, pylorus-preserving gastrectomy; spRRDG, single-port reduced-port robotic distal gastrectomy; TG, total gastrectomy

DG cases were exclusively selected to minimize heterogeneity in surgical extent, lymphadenectomy field, and reconstruction techniques. TG, PG, and PPG differ substantially in operative complexity, LN station clearance, anastomotic configuration, and postoperative recovery profiles. Inclusion of these procedures could introduce confounding effects when comparing surgical platforms. Therefore, restricting the analysis to DG ensured a more homogeneous operative population, allowing for a more reliable assessment of platform-related differences in lymphadenectomy capability, operative efficiency, and postoperative recovery.

### 2.3. Surgical Procedure and Port Placement of Reduced-Port Robotic DG

In accordance with the KGCA practice guidelines, DG was performed for tumors located in the low to mid-body of the stomach, where adequate proximal resection margins could be safely secured. Based on the preoperative clinical staging, standard LND was conducted according to the recommended extent of dissection for clinical (c) T1 or ≥cT2 disease [28]. After the distal stomach was resected, gastrointestinal continuity was restored using Billroth I, Billroth II, or Roux-en-Y reconstruction at the discretion of the operating surgeon.

**Figure 2:**
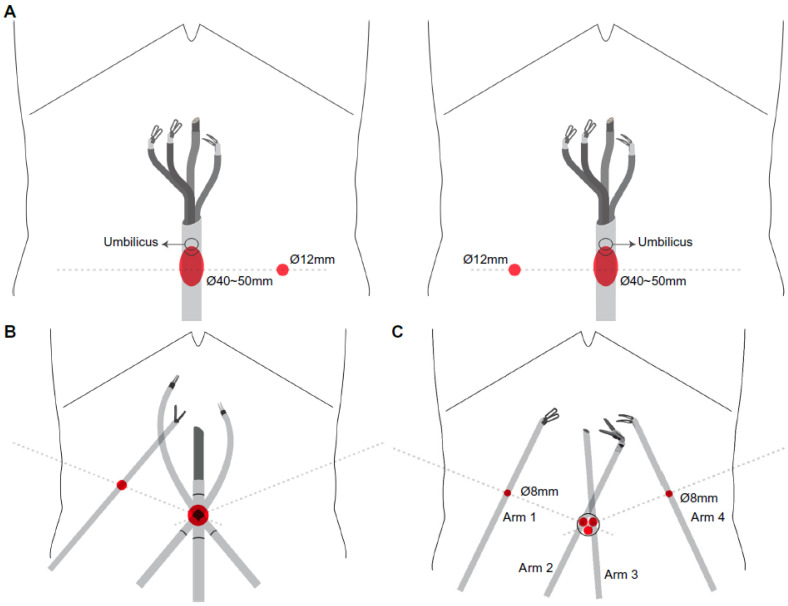
Port configuration of the spRRDG and cRRDG. (**A**) Port configuration for the spRRDG using the da Vinci SP system. A single-port cannula is inserted transumbilically, through which all three articulating robotic instruments and the flexible endoscope are deployed. A 12-mm assist port (red circle) is positioned either on the left or right lower abdomen to facilitate the introduction of energy devices, suction, or laparoscopic staplers when needed. Abbreviations: spRRDG; single-port reduced-port robotic distal gastrectomy; Ø, diameter. (**B**) Port configuration for the cRRDG using the da Vinci Single-Site™ platform. A curved, multi-channel Single-Site umbilical port (central red circle) accommodates a three-dimensional endoscope and two semi-rigid Single-Site instruments arranged in a crossing configuration. Abbreviations: cRRDG, conventional reduced-port robotic distal gastrectomy; Ø, diameter. (**C**) Port configuration of cRRDG using multi-arm robotic platforms.

Port placement details, including the robotic system used, incision sites, and the number of trocars, varied among surgeons. Figure 2 illustrates representative port placements for reduced-port robotic DG (RRDG) with the da Vinci SP (spRRDG) platform and conventional RRDG (cRRDG). In spRRDG, a single-port access was established through an umbilical or Pfannenstiel incision, with one additional 12-mm assist port positioned on the patients’ left- or right-hand sides (Figure 2A). Although one additional 12-mm assistant port was required, the procedure was defined as spRRDG because the lymphadenectomy and principal operative steps were performed using the da Vinci SP platform through a single multi-channel robotic cannula. During the registry period, a dedicated SP-compatible robotic stapler was not commercially available; therefore, gastrointestinal reconstruction required the use of a conventional laparoscopic linear stapler via an auxiliary port. Stapling was conducted by a trained bedside assistant, typically a junior surgeon or clinical fellow, under direct robotic visualization.

In cRRDG, various multi-arm robotic systems, including the da Vinci S, Si, and Xi surgical systems (Intuitive Surgical Inc., Sunnyvale, CA, USA) were used. The da Vinci Single-Site™ was used in some instances (Figure 2B), while a multi-channel umbilical port was used in other instances to accommodate two robotic arms through a single incision, together with two additional 8- or 12-mm ports placed bilaterally in the abdomen, resulting in a total of three incisions (Figure 2C).

A multi-channel umbilical port (central red circle) accommodates two robotic arms introduced through a single incision. Two additional 8-mm lateral ports (red circles) are placed bilaterally in the abdomen to allow insertion of the remaining robotic arms and auxiliary instruments, resulting in a three-incision configuration. Abbreviations: cRRDG, conventional reduced-port robotic distal gastrectomy; Ø, diameter

### 2.4. Data Analyses

Comparative analyses were performed between the spRRDG (*n* = 86) and the cRRDG (*n* = 734) groups. Clinical variables evaluated included baseline characteristics (age, sex, body mass index [BMI], American Society of Anesthesiologists [ASA] classification, history of abdominal surgery), operative outcomes (operative time, estimated blood loss, extent of LND, number of retrieved and metastatic positive LNs [rLNs, pLNs], type of reconstruction), pathologic outcomes (pT and pN stages), and postoperative recovery outcomes (length of hospital stay, time to first flatus, time to initiation of soft diet [SD], and postoperative complications classified using the Clavien–Dindo system [CD]).

Continuous variables were compared using Student’s *t*- or Mann–Whitney U tests, depending on distributional assumptions. Categorical variables were analyzed using χ^2^ or Fisher’s exact tests. Subgroup analyses were performed according to the extent of lymphadenectomy (D1+ vs. D2). All statistical analyses and figure generation were conducted using SPSS (version 29.0.2.0, IBM Corp., Armonk, NY, USA) and GraphPad Prism (version 10.3.0, GraphPad Software, San Diego, CA, USA) software. All tests were two-sided, and *p* < 0.05 was considered statistically significant.

## 3. Results

### 3.1. Patient Characteristics

In total, 820 patients were enrolled following the application of the inclusion criteria, comprising 86 who underwent spRRDG and 734 who had cRRDG using a multi-port system. The baseline clinicopathological characteristics are summarized in Table 1. Mean age did not differ significantly between the spRRDG and cRRDG groups (57.52 ± 1.31 vs. 56.84 ± 0.42 years, *p* = 0.6032). Moreover, the patients’ BMIs were comparable (24.20 ± 0.32 vs. 24.10 ± 0.13 kg/m^2^, *p* = 0.8010). Furthermore, sex distribution (men: 52.3% vs. 56.7%, *p* = 0.4417), history of abdominal surgery (27.9% vs. 23.3%, *p* = 0.3421), and the proportion of patients with an ASA score ≥ 3 (15.1% vs. 17.2%, *p* = 0.6317) were similar between the groups.

### 3.2. Overall Surgical Outcomes

The operative and postoperative outcomes for the overall cohort are presented in Table 2. The spRRDG group demonstrated a significantly longer mean operative time compared with the cRRDG group (227.06 ± 6.19 vs. 183.58 ± 2.18 min, *p* < 0.0001). Additionally, estimated blood loss was significantly higher in the spRRDG group (60.22 ± 8.76 vs. 40.76 ± 2.32 mL, *p* = 0.0088). rLN was significantly lower in the spRRDG group (36.38 ± 1.53 vs. 46.52 ± 0.66, *p* < 0.0001), while the number of pLNs did not differ significantly between the groups (0.30 ± 0.10 vs. 0.78 ± 0.12, *p* = 0.1887). Importantly, the proportion of patients with rLNs ≥ 16 was comparable between the two approaches (97.7% vs. 98.9%, *p* = 0.3233), indicating no significant difference in achieving the conventional adequacy threshold for rLN. The extent of LND (<D2: 79.7% vs. 80.2%, *p* = 0.3695), type of reconstruction (Billroth I: 44.1% vs. 34.9%; Billroth II: 51.6% vs. 61.6%; Roux-en-Y: 4.2% vs. 3.5%, *p* = 0.2135), pathologic T stage (T1: 83.5% vs. 84.9%; T2: 6.9% vs. 10.5%; T3: 5.9% vs. 3.5%; T4: 3.7% vs. 1.2%, *p* = 0.2242), and pathologic N stage (N0: 83.5% vs. 87.2%; N1: 9.4% vs. 7.0%; N2: 3.5% vs. 5.8%; N3: 3.5% vs. 0%, *p* = 0.1937) exhibited no statistically significant differences between the spRRDG and cRRDG groups.

Postoperative recovery parameters favored spRRDG (Table 3), with the approach demonstrating a shorter hospital stay (4.06 ± 0.23 vs. 5.95 ± 0.13 days, *p* < 0.0001), earlier first flatus (postoperative day [POD] 2.24 ± 0.10 vs. 3.08 ± 0.04 days, *p* < 0.0001), and earlier SD initiation (POD 1.59 ± 0.14 vs. 2.89 ± 0.07 days, *p* < 0.0001) compared with the cRRDG approach. The incidence of early complications, using the CD classification, was similar between the two groups (overall: 31.4% vs. 38.1%, *p* = 0.6500). No postoperative mortality was observed in either group.

### 3.3. Subgroup Analysis According to LND Extent

#### 3.3.1. D1+ LND

In the D1+ subgroup (Table 4), operative time was longer in the spRRDG group (219.01 ± 6.47 vs. 178.91 ± 2.51 min, *p* < 0.0001). Estimated blood loss was higher (51.67 ± 7.27 vs. 36.72 ± 2.37 mL, *p* = 0.0386), and fewer lymph nodes were retrieved in spRRDG (34.09 ± 1.58 vs. 44.36 ± 0.72, *p* < 0.0001). The proportion of patients with rLNs ≥ 16 was also comparable between the two groups (97.1% vs. 98.6%, *p* = 0.3608), indicating no significant difference in achieving adequate rLN. However, enhanced recovery after surgery (ERAS)-related outcomes remained more pronounced in the spRRDG group: hospital stay was shorter (4.00 ± 0.27 vs. 5.84 ± 0.15 days, *p* < 0.0001), and first flatus (POD 2.25 ± 0.11 vs. 3.14 ± 0.05 days, *p* < 0.0001) and SD initiation (POD 1.55 ± 0.16 vs. 2.89 ± 0.07 days, *p* < 0.0001) were significantly earlier. Early postoperative complication rates showed no significant differences between the two groups (*p* = 0.4581).

#### 3.3.2. D2 LND

In the D2 subgroup (Table 5), operative time was significantly longer in the spRRDG group than in the cRRDG group (259.71 ± 15.03 vs. 198.29 ± 4.22 min, *p* < 0.0001). Fewer rLNs were observed in the spRRDG group than in the cRRDG group; however, the difference was not significant (45.71 ± 3.69 vs. 53.30 ± 1.39, *p* = 0.1030). All patients in both groups achieved rLNs ≥ 16 (100% vs. 100%), confirming complete compliance with the conventional adequacy threshold for D2 LND. Postoperative recovery parameters favored the spRRDG group, exhibiting shorter hospital stays (4.29 ± 0.32 vs. 6.29 ± 0.30 days, *p* = 0.0382) and earlier first flatus (POD 2.24 ± 0.14 vs. 2.89 ± 0.08 days, *p* = 0.0081). Earlier SD initiation was also observed in the spRRDG group (POD 1.77 ± 0.32 vs. 2.91 ± 0.20 days, *p* = 0.0828), although this trend did not reach statistical significance. Early complication rates were comparable between the groups (*p* = 0.3161).

## 4. Discussion

RRDGs have been performed using multiple versions of the da Vinci robotic surgical systems (Intuitive Surgical) [15,22,23,24,29]. While such procedures have adopted diverse accessories, most are combined with multi-port robotic surgical platforms. However, with the introduction of the da Vinci SP system (Intuitive Surgical) in gastric cancer surgery [23,24,25,27], RRDG is evolving into a new paradigm, where lymphadenectomy is fully covered by the articulating instruments provided via a single cannula. Despite previous studies having emphasized that the articulation was a significant benefit of robot surgery, ≥2 cannulas were established to insert the instruments for lymphadenectomy in RRDG using a conventional system [15,27]. Therefore, to verify the clinical effectiveness of RRDG using da Vinci SP, we compared the short-term outcomes between spRRDG and the conventional approach.

The markedly prolonged operative time observed in the spRRDG cohort compared with that in the cRRDG group is attributable to several intrinsic limitations of the da Vinci SP platform. The unavailability of dedicated energy devices on the SP system primarily impedes the efficiency of tissue dissection and hemostasis, particularly during lymphadenectomy. Additionally, the absence of a robotic stapler necessitates assisted laparoscopic stapling or reconstruction through a constrained assistant port, which may extend the duration of anastomotic procedures [30]. Moreover, the restricted operative field (approximately the size of a tennis ball) substantially limits instrument mobility and spatial access, increasing the complexity and duration of intricate surgical tasks. This spatial limitation prompts frequent camera repositioning, compelling the operator to alternate between above- and below-view modes owing to the ergonomic characteristics of the SP system. The below-view orientation is relatively unfamiliar to most gastric surgeons, necessitating adaptation and potentially prolonging operative delays. These technical and ergonomic challenges possibly drive the longer operative times associated with single-port RG.

Concerning postoperative recovery, the spRRDG group demonstrated a significantly shorter hospital stay, a more rapid return of bowel function (as indicated by earlier first flatus), and an earlier advancement to an SD compared with the cRRDG group, suggesting superior outcomes regarding ERAS protocols. Postoperative pain scores were not included in the present cohort, but previous studies and results from other surgical fields involving the SP system indicate that the enhanced minimal invasiveness by the platform is associated with reduced postoperative pain [31], which may improve ERAS outcomes. The SP system represents the most advanced modality for minimally invasive gastric cancer surgery, and ongoing innovations in robotic instrumentation, such as development of compatible energy devices and staplers, may strengthen its contribution to accelerated postoperative recovery and patient-centered outcomes within ERAS frameworks.

Despite the number of rLNs being significantly lower in the spRRDG group than in the cRRDG group, the mean count exceeded the oncologically acceptable threshold of rLN ≥ 16, ensuring reliable pathological staging. Ooi et al. reported that rLN ≥ 16 was associated with improved pN classification owing to stage migration, while overall survival remained comparable when stratified using LN counts [32]. In the present study, the mean number of rLNs in spRRDG was approximately 34 for LND1+ and >45 for LND2, indicating sufficient LND even with spRRDG. Considering that greater LN improves survival, particularly in intermediate-stage gastric cancer (Stage IIIA) [33], the oncologic adequacy of spRRDG appears to be acceptable for early-stage disease and may be feasible even for selected Stage II cases. However, until further prospective evidence confirms its long-term oncologic safety, the application of spRRDG should preferably be limited to early gastric cancer.

To our knowledge, this study represents the first and largest multicenter comparative analysis of spRRDG and cRRDG for gastric cancer, which considered the da Vinci SP platform and other multi-port robotic platforms. Despite its retrospective design, this study provides foundational evidence supporting the feasibility of SP robotic systems in MIS for gastric cancer.

Nevertheless, this study has some limitations. As a retrospective study involving multiple institutions, inherent biases may exist, including variability in surgical expertise and institutional protocols. The da Vinci SP system was introduced relatively recently for gastric surgery, with most reports emerging in 2022–2023 [23,24,25,27,34]. This short period of adopting spRRDG suggests a relative difference in surgeon proficiency between the SP and conventional systems within this cohort, which may influence outcomes. Finally, owing to the limited adoption period of the SP platform, this study was restricted to short-term surgical outcomes, and long-term oncologic safety could not be evaluated.

Despite these limitations, our findings demonstrate the technical feasibility and perioperative safety of spRRDG in real-world multicenter settings, with a particular advantage in postoperative recovery. While there are potential limitations in the surgical extent of reduced-port gastrectomy [35], the continuous evolution of robotic platforms, particularly with forthcoming developments such as SP-compatible energy devices and staplers, may expand applications, including more complex resections, under a refined reduced-port approach.

## 5. Conclusions

The clinical application of spRRDG should remain limited to early gastric cancer until robust evidence from prospective studies emerges. This approach ensures patient safety and provides an essential foundation for the expansion of single-port robotic techniques in gastric cancer surgery. The present analysis, based on a multicenter real-world cohort, provides meaningful evidence supporting the feasibility and perioperative safety of the da Vinci SP platform in RRDG.

## Figures and Tables

**Table 1:** Baseline characteristics of patients who underwent RRDG for gastric cancer: conventional REPROG versus the da Vinci SP.

Variables	Type of Instrument		*p*-Value
	Conventional REPROG (*n* = 734)	da Vinci SP (*n* = 86)	
Age (years) at operation *	56.84 ± 0.42	57.52 ± 1.31	0.6030
Sex			0.4417
Male (%)	416 (56.7%)	45 (52.3%)	
Female (%)	318 (43.3%)	44 (47.7%)	
BMI *	24.10 ± 0.13	24.20 ± 0.32	0.8010
Abdominal OP history			0.3421
Yes	171 (23.3%)	24 (27.9%)	
No	563 (76.7%)	62 (72.1%)	
ASA score			0.6317
<3	608 (82.8%)	73 (84.9%)	
≥3	126 (17.2%)	13 (15.1%)	

Variables with asterisks (*) are expressed as mean ± standard error. Abbreviations: ASA, American Society of Anesthesiologists; BMI, body mass index; OP, operation; REPROG, reduced-port robotic gastrectomy; SP, single port.

**Table 2:** Surgical outcomes of patients who underwent robotic DG for gastric cancer: conventional REPROG versus the da Vinci SP.

Variables	Type of Instrument		*p*-Value
	Conventional REPROG (*n* = 734)	da Vinci SP (*n* = 86)	
Extent of LND			0.3695
<D2 (%)	557 (79.7%)	69 (80.2%)	
≥D2 (%)	177 (24.1%)	17 (19.8%)	
Type of reconstruction			0.2135
Billroth I (%)	324 (44.1%)	30 (34.9%)	
Billroth II (%)	379 (51.6%)	53 (61.6%)	
Roux-en-Y (%)	31 (4.2%)	3 (3.5%)	
Operation time (min) *	183.58 ± 2.18	227.06 ± 6.19	<0.0001
Amount of bleeding (mL) *	40.76 ± 2.32	60.22 ± 8.76	0.0088
rLN *	46.52 ± 0.66	36.38 ± 1.53	<0.0001
pLN *	0.78 ± 0.12	0.30 ± 0.10	0.1887
rLN ≥ 16	726 (98.9%)	84 (97.7%)	0.3233
pT stage			0.2242
T1	613 (83.5%)	73 (84.9%)	
T2	51 (6.9%)	9 (10.5%)	
T3	43 (5.9%)	3 (3.5%)	
T4	27 (3.7%)	1 (1.2%)	
pN stage			0.1937
N0	613 (83.5%)	75 (87.2%)	
N1	69 (9.4%)	6 (7%)	
N2	26 (3.5%)	5 (5.8%)	
N3	26 (3.5%)	0	

* Variables are expressed as mean ± standard error. Abbreviations: LND, lymph node dissection; pLN, number of positive lymph nodes; pN stage, pathologic N stage; pT stage, pathologic T stage; REPROG, reduced-port robotic gastrectomy; rLN, total number of retrieved lymph nodes; SP, single port.

**Table 3:** Postoperative recovery outcomes of patients who underwent robotic DG for gastric cancer: conventional REPROG versus the da Vinci SP.

Variables	Type of Instrument		*p*-Value
	Conventional REPROG (*n* = 734)	da Vinci SP (*n* = 86)	
Hospital stay (days) *	5.95 ± 0.13	4.06 ± 0.23	<0.0001
Gas out (POD) *	3.08 ± 0.04	2.24 ± 0.10	<0.0001
Start of SD (POD) *	2.89 ± 0.07	1.59 ± 0.14	<0.0001
Early complication			0.6500
No.	454 (61.9%)	59 (68.6%)	
CD Grade I	150 (20.4%)	16 (18.6%)	
CD Grade II	120 (16.3%)	11 (12.8%)	
CD Grade IIIa	5 (0.7%)	0 (0.0)	
CD Grade IIIb	5 (0.7%)	0 (0.0)	
CD Grade IVa	0 (0.0%)	0 (0.0)	
Mortality	0 (0.0)	0 (0.0)	NA

* Variables are expressed as mean ± standard error. Abbreviations: CD, Clavien–Dindo; NA, not applicable; No. number; POD, postoperative day; REPROG, Reduced-port robotic gastrectomy; SD, soft diet; SP, single port.

**Table 4:** Surgical outcomes of standardized patients who underwent robotic DG with D1+ dissection: conventional REPROG versus the da Vinci SP system.

Variables	Type of Instrument		*p*-Value
	Conventional REPROG (*n* = 557)	da Vinci SP (*n* = 69)	
Operation time (min) *	178.91 ± 2.51	219.01 ± 6.47	<0.0001
Amount of bleeding (mL) *	36.72 ± 2.37	51.67 ± 7.27	0.0386
rLN *	44.36 ± 0.72	34.09 ± 1.58	<0.0001
pLN *	0.22 ± 0.05	0.16 ± 0.08	0.6648
rLN ≥ 16	549 (98.6%)	67 (97.1%)	0.3608
Hospital stay (days) *	5.84 ± 0.15	4.00 ± 0.27	<0.0001
Gas out (POD) *	3.14 ± 0.05	2.25 ± 0.11	<0.0001
Start of SD (POD) *	2.89 ± 0.07	1.55 ± 0.16	<0.0001
Early complication			0.4581
No.	350 (62.8%)	50 (72.5%)	
CD Grade I	115 (20.6%)	13 (18.8%)	
CD Grade II	86 (15.4%)	6 (8.7%)	
CD Grade IIIa	3 (0.5%)	0 (0.0)	
CD Grade IIIb	3 (0.5%)	0 (0.0)	
CD Grade IVa	0 (0.0%)	0 (0.0)	

* Variables are expressed as mean ± standard error. Abbreviations: CD, Clavien–Dindo; No., number; pLN, number of positive lymph nodes; POD, postoperative day; REPROG, reduced-port robotic gastrectomy; rLN, total number of retrieved lymph nodes; SD, soft diet; SP, single port.

**Table 5:** Surgical outcomes of standardized patients who underwent robotic DG with D2 dissection: conventional REPROG versus the da Vinci SP system.

Variables	Type of Instrument		*p*-Value
	Conventional REPROG (*n* = 177)	da Vinci SP (*n* = 17)	
Operation time (min) *	198.29 ± 4.22	259.71 ± 15.03	<0.0001
Amount of bleeding (mL) *	53.47 ± 5.98	94.94 ± 32.51	0.0574
rLN *	53.30 ± 1.39	45.71 ± 3.69	0.1030
pLN *	2.54 ± 0.47	0.88 ± 0.35	0.2741
rLN ≥ 16	177 (100%)	17 (100%)	-
Hospital stay (days) *	6.29 ± 0.30	4.29 ± 0.32	0.0382
Gas out (POD) *	2.89 ± 0.08	2.24 ± 0.14	0.0081
Start of SD (POD) *	2.91 ± 0.20	1.77 ± 0.32	0.0828
Early complication			0.3161
No.	104 (58.8%)	9 (52.9%)	
CD Grade I	35 (19.8%)	3 (17.6%)	
CD Grade II	34 (19.2%)	5 (29.4%)	
CD Grade IIIa	2 (1.1%)	0 (0.0%)	
CD Grade IIIb	1 (1.1%)	0 (0.0%)	
CD Grade IVa	0 (0.0%)	0 (0.0)	

* Variables are expressed as mean ± standard error. CD, Clavien–Dindo; No., number; pLN, number of positive lymph nodes; POD, postoperative day; REPROG, reduced-port robotic gastrectomy; rLN, total number of retrieved lymph nodes; SD, soft diet; SP, single port.

## Data Availability

The data presented in this study are available on request from the corresponding author.

## Abbreviations

The following abbreviations are used in this manuscript:

ASA	American Society of Anesthesiologists
BMI	body mass index
CD	Clavien–Dindo system
cRRDG	conventional reduced-port robotic distal gastrectomy
DG	distal gastrectomy
KGCA	Korean Gastric Cancer Association
KLASS-13	Korean Laparoendoscopic Gastrointestinal Surgery Study-13
LG	laparoscopic gastrectomy
LND	lymph node dissection
MIS	minimally invasive surgery
POD	postoperative day
REPROG	reduced-port robotic gastrectomy
RG	robotic gastrectomy
RRDG	reduced-port robotic distal gastrectomy
SD	soft diet
spRRDG	da Vinci SP reduced-port robotic distal gastrectomy
